# Daily online adaptation enhances target coverage in prostate cancer radiotherapy: a retrospective analysis

**DOI:** 10.3389/fonc.2025.1662671

**Published:** 2025-11-03

**Authors:** Hanna Malygina, Bryan Salazar Zuniga, Hendrik Auerbach, Marc Ries, Yvonne Dzierma, Markus Hecht, Jan Palm

**Affiliations:** Department of Radiotherapy and Radiation Oncology, Saarland University Medical Center, Homburg, Germany

**Keywords:** prostate cancer, online adaptive radiotherapy (oART), Varian Ethos, dosimetric impact, dosimetric distribution, organs-at-risk sparing, CBCT

## Abstract

**Introduction:**

Online adaptive radiotherapy aims to improve treatment quality by accounting for inter-fractional variation in anatomy. This study presents a quantitative comparison between adapted and non-adapted scheduled plans with identical margins in a real-world clinical setting.

**Methods:**

We retrospectively analyzed 422 fractions from 43 patients with prostate cancer treated with the Varian Ethos system. All patients received hypofractionated treatment with 3 Gy per fraction up to a cumulative dose of 60 Gy. For each fraction, the scheduled plan (planned on planning CT, calculated on synthetic CT derived from daily cone beam CT) was compared to the adapted plan (planned and calculated on actual daily anatomy) by means of several dose-volume metrics. Comparative statistics regarding dose-volume metrics were performed using Wilcoxon signed-rank test for paired data with a two-sided hypothesis.

**Results:**

Adapted plans delivered significantly better target coverage, conformality, and homo-geneity than scheduled plans. The constraints D95% ≥ 95% and V95% ≥ 95% were met in 418 out of 422 fractions with the adapted plan, compared to only 41%-84% of fractions with the scheduled plan. Median absolute improvements for these metrics ranged between 1.5 and 6.0 percentage points. Most organ-at-risk metrics remained unchanged or showed only minor differences. Interquartile ranges decreased across all metrics.

**Conclusions:**

Adaptation significantly improved target dose metrics compared to non-adapted plans, without compromising organs-at-risk sparing. Interquartile ranges were reduced for all metrics evidencing better repeatability of adapted plans.

## 1 Introduction

Prostate cancer is among the most common malignancies affecting men worldwide (1, 2), and radiotherapy (RT) remains one of its cornerstone treatment modalities. However, daily anatomical variations—particularly in bladder and rectal filling—pose a significant challenge to accurate dose delivery. Such interfractional changes can lead to undercoverage of the prostate target and unintended dose escalation to surrounding organs at risk (OARs), thereby compromising tumor control and increasing the risk of treatment-related toxicity (3, 4).

Online adaptive radiotherapy (oART) has emerged to address these challenges by enabling real-time modification of the treatment plan based on each day’s patient anatomy. By acquiring a cone-beam CT (CBCT) on each treatment day, re-segmenting targets and OARs, and re-optimizing the dose distribution, oART can substantially mitigate the effects of anatomical variability and enhance treatment precision.

The Varian Ethos system (Varian Medical Systems, Palo Alto, CA, USA) (5) integrates daily CBCT imaging with artificial-intelligence-driven auto-segmentation and fully automated plan re-optimization, creating a seamless workflow for oART in routine clinical practice. This capability is particularly valuable in the management of prostate cancer, where bladder and rectal filling can induce significant prostate motion. In hypofractionated regimens—where each fraction delivers a high dose per session—such precision is critical. Daily adaptation not only improves target coverage but also holds the promise of reducing toxicity to the bladder, rectum, and other pelvic structures.

Several studies have shown dosimetric benefits of adaptation for a limited number of patients (partially with simulated data) for different prostate cases: prostate stereotactic body radiation therapy (6), prostate bed (7), prostatic fossa (8), and prostate and seminal vesicles (9). The advantages of oART were also reported for gynecological (10), rectal (11), bladder (12, 13), and other cancers. In this study, we present a large and consistent cohort of 40 prostate cancer patients who underwent oART using the Varian Ethos system with a double simultaneous integrated boost (SIB) technique at our department.

## 2 Method and materials

### 2.1 Online adaptive radiotherapy workflow

CBCT-based oART using the Varian Ethos system is conducted with a pre-defined workflow. The process begins with the planning CT (pCT), where the treatment intent—including dose prescription, planning objectives, and delineation of OARs—is established.

A reference treatment plan is generated on the planning CT using one of several predefined beam configurations: intensity-modulated radiotherapy (IMRT) with 7, 9, or 12 equidistant fields, or volumetric modulated arc therapy (VMAT) with two or three arcs. Once this reference plan is approved, it becomes available for daily treatment. Our early clinical experience indicated that VMAT plan calculation required considerably more time while offering only marginal dosimetric benefit compared with IMRT. For this reason, VMAT plans (Ethos 1.0) were not used in routine clinical practice at our institution.

**Figure 1:**
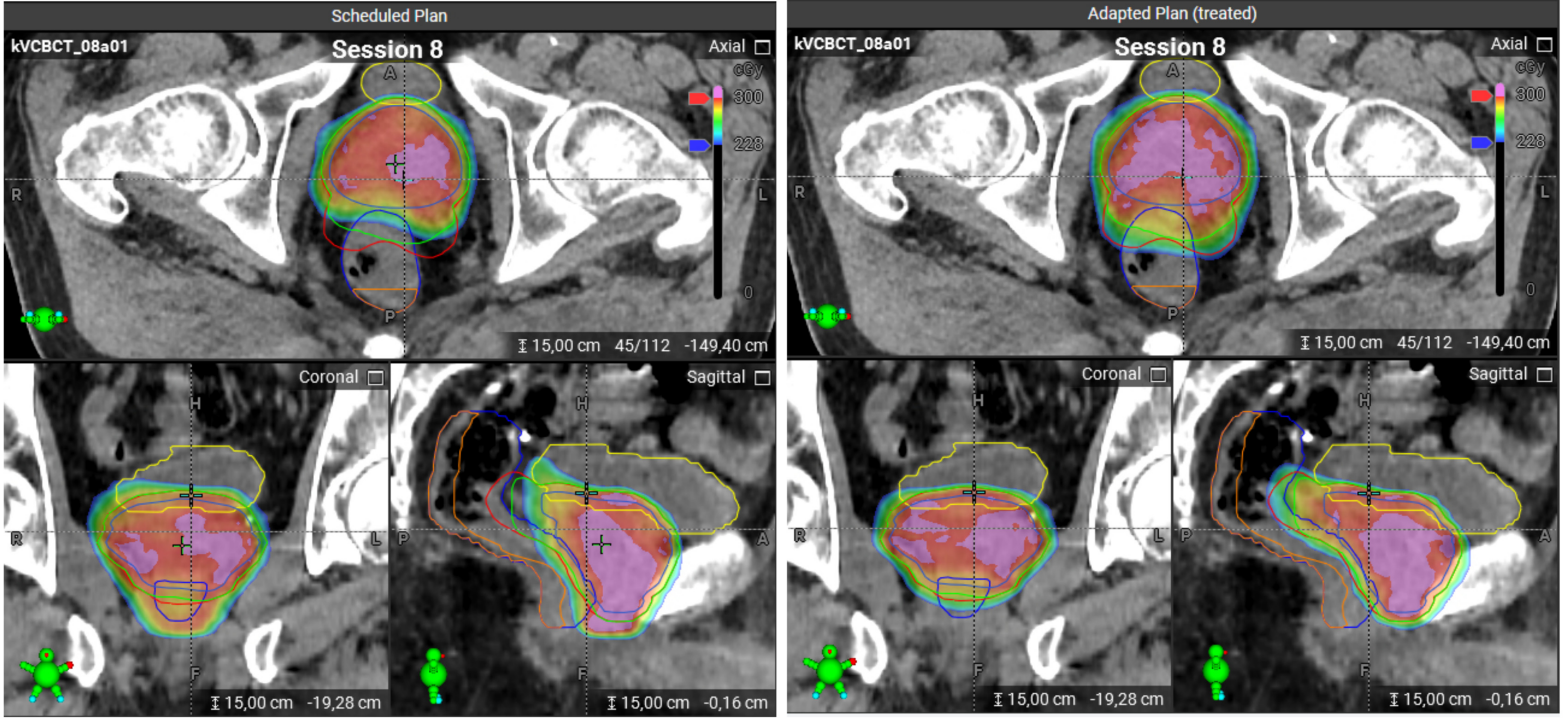
Scheduled and adapted plans on a CBCT image for the same treatment session. Left panel: The scheduled plan. Right panel: The adapted plan. The color scheme for the contours: bladder – yellow, rectum – dark blue, PRW – orange, PTV/SIB1/SIB2 – red/green/blue. The dose distributions are visualized using a color wash, where blue corresponds to 2.28 Gy and red to 3 Gy. Doses above 3 Gy are indicated in pink. The scheduled plan shows strong underdosage for PTV and SIB1 which could be compensated with the adapted plan, as can be seen in sagittal and axial views.

At each treatment session, the patient is positioned and a CBCT scan is acquired. Following a quality check of the image, the system automatically propagates the planning contours to the CBCT of the day. These propagated contours must then be reviewed and, if necessary, edited by the user. Using deformable image registration, the CBCT anatomy is mapped back to the planning CT to preserve Hounsfield unit accuracy (synthetic CT). On this basis, two dose distributions are calculated: (1) the dose from the scheduled (non-adapted) plan applied to the current anatomy, and (2) a newly re-optimized adapted plan, generated using the original treatment intent and constraints, tailored to the anatomy of the day (**Figure 1**).

The clinician then compares both plans and selects the one to be delivered. In practice, the adapted plan typically offers superior dosimetric quality, and at our institution it is selected in *>* 99% of sessions for treatment.

### 2.2 Treatment characteristics

Between July 2023 and October 2024, a total of 72 patients were treated with the Ethos system at our institution. The majority of patients underwent pelvic radiotherapy, primarily for prostate cancer. Patients with primary prostate cancer radiotherapy are treated at our institution with the in-house protocol based on the CHHiP trial (14).

For this *post-hoc* analysis, we selected all patients with a confirmed diagnosis of prostate cancer, who were treated with the 2 SIBs concept at our institution and whose data could be fully exported from the Ethos system. These 49 patients had been treated prior to the commencement of this study, making this an exploratory analysis.

Planning target volume (PTV), SIB1, and SIB2 are structures derived from prostate and seminal vesicles contours, which is necessary for the adaptive treatment workflow since they will be automatically generated by the system based on the adapted prostate and seminal vesicle contours. SIB2 is defined as the prostate with 3 mm margins (posteriorly 0 mm). SIB1 includes the prostate and the proximal 1 cm of the seminal vesicles with 6 mm margins (posteriorly 3 mm). PTV is defined the same as SIB1 but includes the proximal 2 cm of the seminal vesicles with 6 mm margins in all directions including posterior. The cumulative prescribed doses for PTV, SIB1, and SIB2 are respectively 48 Gy, 57.6 Gy, and 60 Gy.

Dose objectives for OARs in this study were aligned with our institution’s in-house protocol (15), which is based on the guidelines from the CHHiP (14), PROFIT (16), PACE-B (17), and PACE-C (18) trials. In our institution, a posterior rectum wall (PRW) is used as an additional OAR (reasoning and PRW contouring have been described previously (19)).

To ensure better bladder sparing, the patients are instructed to follow our in-house “Bladder and bowel preparation instructions” (15), which aim at a reproducibly empty rectum and a comfortably full bladder.

### 2.3 Patient selection

As previously discussed (19), a systematic bias exists in which prostate contours on the pCT tend to be smaller than those on CBCT. This discrepancy does not indicate an error but arises from the ESTRO ACROP contouring guidelines (20), which recommend assuming equal levator ani muscle thickness adjacent to the prostate and rectum on CT, while on MRI (magnetic resonance imaging) these structures can be clearly distinguished. Consequently, MRI-based contouring yields smaller target volumes by avoiding unnecessary inclusion of the levator ani muscles. In CT-only workflows, the Santorini plexus is also frequently included due to limited soft-tissue contrast, further enlarging prostate, CTV, and PTV volumes.

At our institution, MRI is used to support pCT contouring but is not always referenced during adaptive workflows, occasionally leading to larger prostate contours in adapted datasets. Large discrepancies between the prostate contour volume on the pCT (used for the scheduled plan) and on the CBCT (used for the adapted plan) can introduce artifacts in dosimetric comparison (19).

**Figure 2:**
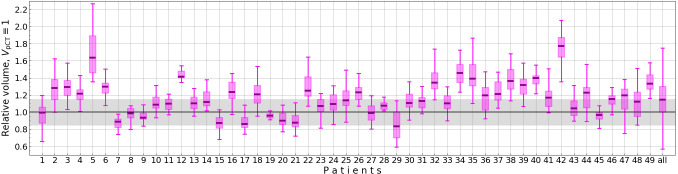
Relative prostate volume on CBCTs (*V*_CBCT_ */V*_pCT_) for each patient. The gray area marks the allowed prostate volumes for a fraction to be included in the study.

To minimize variability and enhance data homogeneity, we applied a threshold of 15% to the pro-state volume for each session: $\Delta V=\left|V_{prostate,pCT}-V_{prostate,CBCT}\right|\leq 15\%.$ This threshold allows to homogenize the data while accounting for physiological prostate swelling often observed during the radiotherapy (21). Fractions exceeding this threshold were excluded, resulting in the removal of 555 out of 980 fractions due to pronounced contour discrepancies (see **Figure 2** for prostate volume distributions). The excluded fractions were analyzed separately. Consequently, six patients were entirely excluded from the study.

Additionally, three interrupted sessions were excluded. The final dataset comprised 422 fractions (ranging from 1 to 20 fractions per patient) from a total of 43 patients, providing a consistent basis for analysis. Among these 422 fractions, the scheduled plan was selected for treatment in only three sessions.

### 2.4 Data analysis

Since this is a retrospective study, all data was available prior to the beginning of the study. Dose and structure DICOM files were exported from the Ethos system. Dose-volume histograms were computed using a custom-developed Python script based on the dicompyler-core package (version 0.5.6) (22).

For each dose-volume metric, we calculated the difference between the metric value obtained with the scheduled plan and that obtained with the adapted plan for each fraction. To assess the significance of these differences, we applied the Wilcoxon signed-rank test for paired data with a two-sided alternative hypothesis.

Additionally, we evaluated the homogeneity index $HI=\left(D1\%-D99\%\right)/D_{prescribed}$ (23) for SIB2 as well as the conformation number CN=TV95%/TV×TV95%/V95% for PTV, where TV95% and V95% are respectively the volume of PTV and the volume of tissue covered by 95% of the PTV prescribed dose, *TV* is the total volume of PTV (24). The CN quantifies both the target coverage (the first term of the formula) and the healthy tissue sparing (the second term).

To estimate both the central tendency and dispersion of non-normally distributed data, we calculated the Hodges-Lehmann median along with the corresponding quartiles (Q1 and Q3).

A custom Python script was developed for this analysis, utilizing core libraries such as NumPy, SciPy, and statistics. Given the exploratory nature of this study, *p*-values are considered descriptive, with *p<*0.05 interpreted as indicative of statistical significance. No Bonferroni correction was applied; instead, we always present an absolute *p*-value if *p* ≥ 0.001.

## 3 Results

### 3.1 Patient characteristics

A total of 43 patients (**Table 1**) with a confirmed diagnosis of prostate cancer were included in this study. Clinical staging revealed that 24 patients (55.8%) had T1 tumors, 18 (41.9%) had T2 tumors, and 1 patient (2.3%) was classified as T3. Androgen deprivation therapy was administered to 17 patients, depending on clinical indications and risk stratification. Adaptive radiotherapy was delivered in most cases using IMRT techniques. Most patients received either 9-beam or 12-beam IMRT; four patients were treated with different IMRT beam arrangements in different sessions, and one patient received either VMAT or IMRT, although all VMAT-treated fractions were excluded by the prostate-volume criterion.

**Table 1:** Demographic characteristics including age, NCCN (National Comprehensive Cancer Network) risk group, Gleason and ISUP (International Society of Urological Pathology) grades, cancer stage, receiving of the androgen deprivation therapy, the latest iPSA value before or shortly after the start of the treatment, as well as the plan modality.

Age, years	Mean	Min - max
	72.3	55 - 83
NCCN riskgroup	# of patients	% of patients
Low	12	27.9
Intermediate	27	62.8
High	4	9.3
Gleason (ISUP) grade	# of patients	% of patients
6 (1)	12	27.9
7a (2)	18	41.9
7b (3)	9	20.9
8 (4)	3	7.0
Cancer stage	# of patients	% of patients
T1b	1	2.3
T1c	23	53.5
T2a	2	4.7
T2b	2	4.7
T2c	14	32.6
T3	1	2.3
Androgendeprivation therapy	# of patients	% of patients
	17	39.5
iPSA, ng/ml	Mean	min - max
	6	0.08 - 27
Plan	# of patients	% of patients
IMRT 09	12	27.9
IMRT 12	26	60.5
Combination	5	11.6

Some patients received different plan modalities in different sessions (represented by “Combination”).

### 3.2 Target metrics

**Figure 3:**
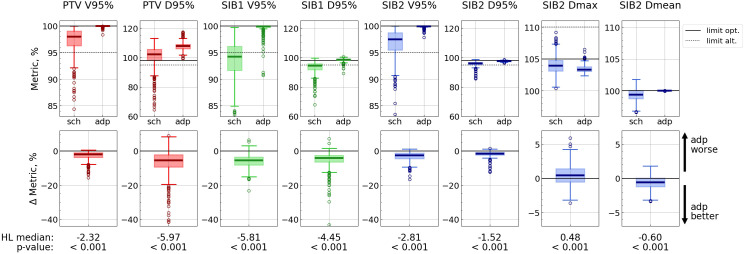
Target metric distributions for scheduled (“sch”) and adapted (“adp”) plans (top panel), and distributions of difference: metric_sch_ − metric_adp_ (bottom panel). Each vertical pair of subplots corresponds to a single metric. Solid lines correspond to optimal limits for each metric, and dotted lines – to alternative ones (top panel). Hodges-Lehmann median for each difference distribution is given under the corresponding subplot, as well as the *p*-value from the corresponding Wilcoxon test. The labels “adp better” and “adp worse” are valid for all metrics except SIB2 Dmax.

Adaptation significantly enhanced target coverage as measured by D95% (*p<*0.001) for all targets, with the improvement ranging from 1.5 to 6.0%. (Hereafter, we estimate metric changes in terms of Hodges-Lehmann median of the difference distributions, all values refer to absolute dose changes, e.g. percentage points.) Additionally, V95% increased on average by 2.3 to 5.8% (**Figure 3**; **Supplementary Table S1** in the **Supplementary Material**). Furthermore, the interquartile range (IQR = Q3 - Q1) decreased with adaptation for all target metrics.

**Figure 4:**
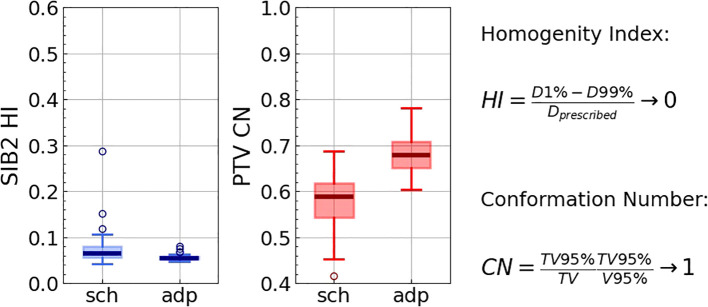
Homogeneity index for SIB2, as well as conformation number for PTV for all 422 fractions for scheduled (“sch”) and adapted (“adp”) plans. The right panel presents the definitions and the ideal values for the indices.

The adapted plan demonstrated markedly improved homogeneity and conformality (**Figure 4**). Specifically, the homogeneity for SIB2 (ideal value of 0) improved from 0.092[0.080, 0.115] (Hodges-Lehmann median [Q1, Q3]) to 0.056[0.054, 0.059] (*p<*0.001), with its maximum value decreasing from 0.75 to 0.11. The conformation number for PTV (ideal value of 1) increased from 0.66[0.64, 0.68] to 0.685[0.671, 0.699] (*p<*0.001), with its minimum value increasing from 0.53 to 0.62.

For the adapted plan, all alternative target constraints (except SIB2 Dmean) were satisfied in 418 out of 422 fractions: *D*95%≥ 95% and *V*95%≥ 95%. Only in four fractions did both SIB1 constraints fail, while those for SIB2 and PTV were consistently met (**Table 2**).

**Table 2:** The percentage of fractions with satisfied alternative goal (which is equal to the optimal goal for some metrics) for each metric for three categories of the prostate contour volume on CBCT scan: 1. much smaller than on pCT: *V < *0.85*V*_pCT_; 2. within the selected threshold (e.g. included into the main analysis); 3. much bigger than on pCT: *V > *1.15*V*_pCT_.

Metric and alternative goal		Percentage of fractions with satisfied alternative goal					
		With scheduled plan			With adapted plan		
		*V <* 0.85*V*_pCT_	0.85−1.15*V*_pCT_	*V >* 1.15*V*_pCT_	*V <* 0.85*V*_pCT_	0.85−1.15*V*_pCT_	*V >* 1.15*V*_pCT_
PTV	V95% ≥ 95%	91.4	84.4	49.1	100.0	100.0	100.0
PTV	V95% ≥ 95%	91.4	84.4	48.9	100.0	100.0	100.0
SIB1	V95% ≥ 95%	85.7	40.8	2.3	97.1	99.1	99.8
SIB1	D95% ≥ 95%	85.7	40.8	2.3	97.1	99.1	99.8
SIB2	V95% ≥ 95%	97.1	80.6	24.3	100.0	100.0	100.0
SIB2	D95% ≥ 95%	97.1	80.6	24.3	100.0	100.0	100.0
SIB2	Dmean≥ 100%	25.7	27.3	8.5	42.9	65.6	69.7
SIB2	Dmax ≤ 110%	100.0	100.0	99.8	100.0	100.0	100.0
Bladder	V60Gy < 5%	88.6	87.2	90.9	98.6	97.6	94.8
Bladder	V48Gy < 25%	78.6	91.2	95.7	97.1	97.9	95.7
Bladder	V40Gy < 50%	95.7	99.3	99.2	100.0	99.5	99.2
Rectum	V56Gy < 25%	100.0	100.0	100.0	100.0	100.0	100.0
Rectum	V52Gy < 30%	100.0	100.0	100.0	100.0	100.0	100.0
Rectum	V48Gy < 35%	100.0	100.0	100.0	100.0	100.0	100.0
Rectum	V40Gy < 50%	100.0	100.0	100.0	100.0	100.0	100.0
Rectum	V32Gy < 51%	92.9	97.2	99.2	100.0	100.0	100.0
Rectum	V24Gy < 70%	97.1	96.7	95.3	100.0	100.0	99.8
PRW	V37Gy < 5%	84.3	86.0	93.8	100.0	98.8	99.6
PRW	Dmax < 2.1 Gy	52.9	69.2	84.1	91.4	92.4	97.1
Bowel	V48Gy < 6 ccm	25.7	33.4	26.2	27.1	32.9	29.9
Bowel	V40Gy < 17 ccm	42.9	43.6	34.4	38.6	41.7	37.7
Bowel	Dmax < 2.6 Gy	92.9	89.1	91.5	94.3	90.0	91.3

### 3.3 OAR metrics

**Figure 5:**
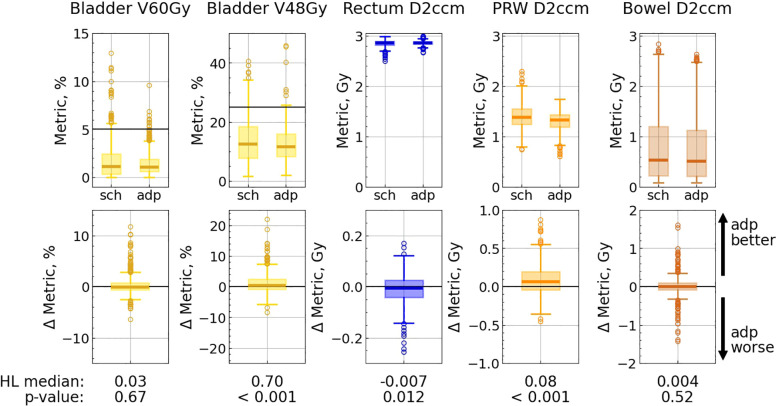
OAR metric distributions for scheduled (“sch”) and adapted (“adp”) plans (top panel), and distributions of difference: metric_sch_ − metric_adp_ (bottom panel). Each pair of subplots corresponds to a single metric. Solid lines correspond to optimal limits for each metric (top panel). Hodges-Lehmann median for each difference distribution is given under the corresponding subplot, as well as the *p*-value from the corresponding Wilcoxon test.

Bladder V60Gy remained unchanged with adaptation, whereas V48Gy and V40Gy exhibited modest but statistically significant (*p<* 0.001) improvements (**Figure 5**; **Supplementary Table S1** in the **Supplementary Material**). The percentage of fractions meeting the optimal constraints for the bladder metrics was higher with the adapted plan than with the scheduled one, and was ranging between 97.6% and 99.5% (**Table 2**).

Among the evaluated rectum metrics (V56Gy, V52Gy, V48Gy, V40Gy, V32Gy, V24Gy, D2ccm), five showed statistically significant changes: the first four metrics experienced a slight deterioration (less than 0.8%) with adaptation, while V24Gy showed a minor improvement. Nevertheless, the adapted plan met all optimal rectum constraints in all fractions (**Table 2**; **Supplementary Table S1** in the **Supplementary Material**).

Furthermore, the adapted plan outperformed the scheduled plan in terms of the PRW metrics: the dose to 2 ccm decreased by 0.08 Gy, the maximum dose was reduced by 0.17 Gy, and V37Gy improved by 0.65% (in all three cases *p<*0.001), while the percentage of fractions meeting the optimal constraint increased for V37Gy from 86% (with the scheduled plan) to 99% (with the adapted one), and for Dmax from 69% to 92% (**Table 2**; **Supplementary Table S1** in the **Supplementary Material**).

The IQR decreased with adaptation for all bladder, rectum, and PRW metrics.

Bowel metrics did not exhibit any significant differences with adaptation.

### 3.4 Excluded sessions

When the prostate contour on CBCT exceeded the 15% threshold (e.g. a bigger prostate on CBCT, 485 sessions), median reductions in the target metrics D95% and V95% ranged from 4.5% to 14.9% (**Supplementary Figure S1** in the **Supplementary Material**). The scheduled plan could not account for such a big prostate on the daily CBCT satisfying the goals for these target metrics in much fewer sessions in comparison with the adapted plan (see **Table 2**). For the sessions with a smaller prostate on CBCT (70 sessions), the adapted plan still conferred statistically significant dosimetric improvements over the scheduled plan, although the magnitude of benefit was reduced (between 0.8% and 2.3%) relative to the cases with a prostate volume close to *V*_pCT_ (**Supplementary Figure S2** in the **Supplementary Material**). The scheduled plan could also fulfill the goals in the most sessions.

Moreover, an enlarged prostate contour on CBCT (and hence larger targets) artificially favored the scheduled plan for OAR metrics—they appeared slightly lower than with adaptation (**Supplementary Figure S1** in the **Supplementary Material**), whereas the converse held true for a smaller prostate contour (**Supplementary Figure S2** in the **Supplementary Material**).

## 4 Discussion

To enable an unbiased comparison between scheduled and adapted plans, we applied a strict exclusion criterion based on prostate contour volume. The rationale for this approach was to avoid artifacts that arise when the prostate contour on the CBCT deviates substantially from that on the planning CT (19). The main reason for this deviation is the availability of MRI fusion for the planning CT but the lack of MRI fusion for daily CBCTs during adaptive sessions. This aspect combined with the ESTRO ACROP contouring guidelines (20), can introduce contouring bias. Naturally, MRI-guided radiotherapy can largely eliminate this issue by providing consistent MRI-based contours for all fractions.

We observed the following artifacts when the prostate appeared larger on CBCT (see columns “*V >* 1.15*V*_pCT_” in **Table 2**; **Supplementary Figure S1** in the **Supplementary Material**):

1. The scheduled plan on CBCT, evaluated using the adapted contours, appears to provide poorer target coverage even in the absence of anatomical changes. This occurs simply because the apparently larger CTV/PTV is not fully encompassed by the prescribed isodose. This does not necessarily mean that the scheduled plan would have been clinically inferior if the prostate volume had been closer to that on the planning CT; however, this artifact artificially amplifies the apparent difference between scheduled and adapted plans.
2. Conversely, the scheduled plan (on either the pCT or CBCT) appears to offer better bladder and rectum sparing, particularly for high-dose metrics, due to the smaller target volume.

For the opposite case (*V*_CBCT_< 0.85*V*_pCT_), the main artifact was worse OAR sparing in the scheduled plan, again as a direct consequence of relatively larger target volumes (see columns “*V <* 0.85*V*_pCT_” in **Table 2**; **Supplementary Figure S2** in the **Supplementary Material**).

Importantly, adaptation maintained high rates of goal satisfaction across all three prostate-volume rangesfor nearly every metric (see **Table 2**). In contrast, the quality of the scheduled plan depended strongly on the relative change in prostate contour volume between pCT and CBCT. For example, for the bigger prostate on CBCT, the scheduled plan showed extremely low percentage of sessions with satisfied goals, going down to only 2.3% for the SIB1 goals.

Combining all prostate volume ranges into a single analysis would obscure true effects due to opposing OAR trends: the adapted plan appears superior when *V*_CBCT_ < 0.85*V*_pCT_ but inferior when *V*_CBCT_ *>* 1.15*V*_pCT_. For target metrics, however, the adapted plan consistently outperformed the scheduled one, with prostate volume deviations affecting only the magnitude, not the direction, of the benefit.

Thus, we applied this exclusion criterion to ensure that only genuine anatomical changes between pCT and CBCT were captured, avoiding distortions caused by contour volume discrepancies. We emphasize that consistent contouring is essential for a fair and unbiased assessment of the benefit of oART. Importantly, these inconsistencies influence only the comparison between scheduled and adapted plans and do not compromise actual treatment quality, provided that CBCT contours are anatomically accurate.

We showed that even for the homogenized dataset, the adapted plan yielded statistically significant and markedly superior target coverage compared to the scheduled plan. OAR sparing, in terms of median values for the dose metrics, was comparable between the scheduled and the adapted plans, although some OAR metrics exhibited statistically significant differences. This outcome is expected given the prioritization schema in our treatment planning system: target V95% metrics along with SIB2 Dmax and Dmean are assigned the highest priority (Priority 1), whereas most OAR metrics are designated as Priority 2 (except Bowel and PRW Dmax, which are also Priority 1). We consider the observed statistically significant differences in OAR metrics to be clinically irrelevant. However, the percentage of fractions meeting the optimal constraints increased notably for bladder and PRW metrics with adaptation.

Our findings are in line with those reported in (6), where the authors analyzed prostate cancer patients treated with stereotactic body RT on a Varian Ethos system. They observed significant improvement for the target metrics, however, the results for OARs were more variable: while the maximum dose to the rectum (represented by D0.03ccm) decreased, it increased for the bladder, and remained unchanged for the sigmoid and bowel. Similarly consistent improvement for the targets but inconsistent effects on OAR have been reported in (7). In their retrospective analysis of 198 fractions from prostate bed patients treated on the Varian Ethos system, a reduction in the IQR was observed for all metrics, which aligns with our results. Smaller IQR indicates high repeatability of dose delivery with the adapted plan.

Comparable outcomes—substantial improvements for targets with limited or variable benefits for OARs—have also been reported for oART in vulvar (10), rectal (11), and pancreatic cancer (25). However, it was shown for 8 patients with pancreatic cancer that adaptation can be statistically significantly beneficial not only for the target but also for most OARs—if the OARs are prioritized over target coverage (26), or at least have the same priority level (27).

In (28) the benefits of adaptation were shown for 3 patients with gastric mucosa-associated lymphoid tissue lymphoma: the adapted plan showed better target coverage and decreased mean dose to liver and kidneys. Thus, both the target and the OARs benefited from the adaptation.

Moreover, even excluding the fractions with high difference in prostate volume relative to the pCT (which can cause among other effects also an artificial underdosage for targets with scheduled plans), we observed occasional instances of low dose coverage for PTV and SIB1 with scheduled plans (*D*95% < 80%). This finding further underscores the importance of oART. The results align with results from (6), where a low PTV coverage sporadically occurred, despite the rigid registration of the CTVs to the CBCT, which ensured consistent CTV volumes between the pCT and each CBCT.

Plan quality in terms of homogeneity and conformality was significantly better for the adapted plan. However, the difference between the adapted and the scheduled plans was not so drastic as reported in (29), where 15 patients with bladder cancer were retrospectively analyzed. The difference between our results though could be explained by the field geometry. In our clinic, an IMRT with a fixed number of fields (mostly 9 or 12) is preferred, while in (29) 3 arc VMAT was utilized. On the other hand, in (30) IMRT was used, and CN values were comparable with those reported in (29). However, both (29) and (30) analyzed bladder cancer patients in contrast to our study with prostate patients.

It is important to note that the comparison of adapted vs. scheduled plans should not be directly interpreted as a comparison between oART and conventional non-adaptive RT. In conventional RT, larger CTV-to-PTV margins are typically employed to maintain target coverage at the cost of OAR sparing: in (8), the authors compared the scheduled plan with larger margins against the adapted plan with reduced margins for postoperative prostate patients. They indeed showed that the tighter margins with adaptation still could provide at least as good coverage as conventional IGRT, furthermore, they led to significantly better OAR sparing. Another study (9) proved the benefits of oART with smaller margins (in comparison to IGRT with conventional margins) for prostate cancer patients for both targets and health tissues (presented by the dose to the body). Similar observations were made for bladder cancer (30) and gynecological cancers (31, 32).

We acknowledge that comparing adapted and scheduled plans on the anatomy of the daily CBCT does not fully reflect the true dosimetric advantages of adaptation, as additional anatomical changes (e.g., bladder filling or bowel gas motion) may occur during the adaptation process and influence the target coverage and OARs sparing (9, 10). In the Ethos system, it is possible to acquire a verification CBCT after adaptation but before treatment delivery. A more realistic dosimetric assessment would require contouring the targets and OARs on this verification CBCT and recalculating the dose-volume metrics. We have previously performed this analysis for 8 patients (19) and highlighted that the “delivered dose” provided by Ethos is of limited value, as it relies on rigid contour propagation. Thus, this delivered dose, although quickly accessible, is not suitable for the realistic dosimetric comparison.

In this study, we demonstrate that even with reduced margins, optimal target coverage is achievable with oART, while still providing equal OAR sparing in comparison with non-adapted plans (with the same margins).

## 5 Conclusions

This study demonstrates that online adaptive radiotherapy provides substantial improvements in target coverage in a clinically realistic setting. Adapted plans consistently achieved better homogeneity and conformality meeting target coverage constraints in nearly all adapted fractions, while OAR sparing stayed the same and the observed differences were not clinically relevant. The reduction in interquartile ranges across dose metrics further highlights the robustness and reproducibility of oART. These findings confirm that oART enables high-quality, consistent treatment delivery and reinforces its value in routine clinical practice for prostate cancer.

## Data Availability

The data analyzed in this study is subject to the following licenses/restrictions: Research data are stored in an institutional repository and will be shared upon request. Requests to access these datasets should be directed to hanna.malygina@uks.eu.
