# Supplementary material for: Daily online adaptation enhances target coverage in prostate cancer radiotherapy: a retrospective analysis
Source: Front Oncol. 2025 Nov 3;15:1662671. doi: 10.3389/fonc.2025.1662671 (PMC12620223; doi:10.3389/fonc.2025.1662671)
Supplement: Supplementary file 1 [file DataSheet1.pdf]

Supplementary Material

1 EXCLUDED FRACTIONS

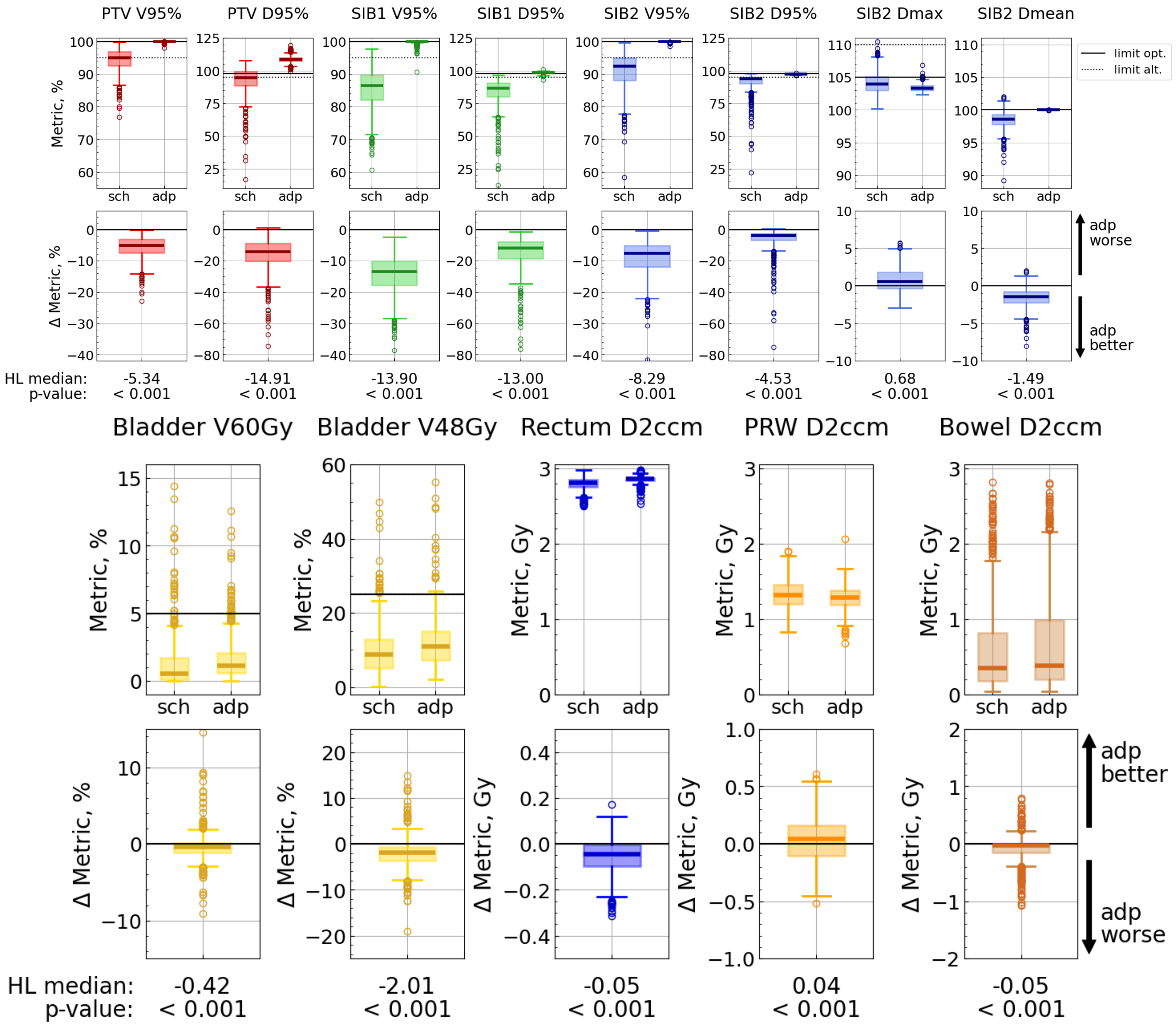

Figure S1. For the fractions with prostate contour **bigger**, than the selected threshold $V_{\mathrm{CBCT}} > 1.15 V_{\mathrm{pCT}}$: Target and OAR metric distributions for scheduled (”sch”) and adapted (”adp”) plans (top panels), and distributions of difference: $\mathrm{metric}_{\mathrm{sch}} - \mathrm{metric}_{\mathrm{adp}}$ (bottom panels). Each pair of subplots corresponds to a single metric. Solid lines correspond to optimal limits for each metric, and dotted lines – to alternative ones (top panels). Hodges-Lehmann median for each difference distribution is given under the corresponding subplot, as well as the p-value from the corresponding Wilcoxon test. The labels ”adp better” and ”adp worse” are valid for all metrics except SIB2 Dmax. Note the different scale for some subplots in comparison with Fig. S2.

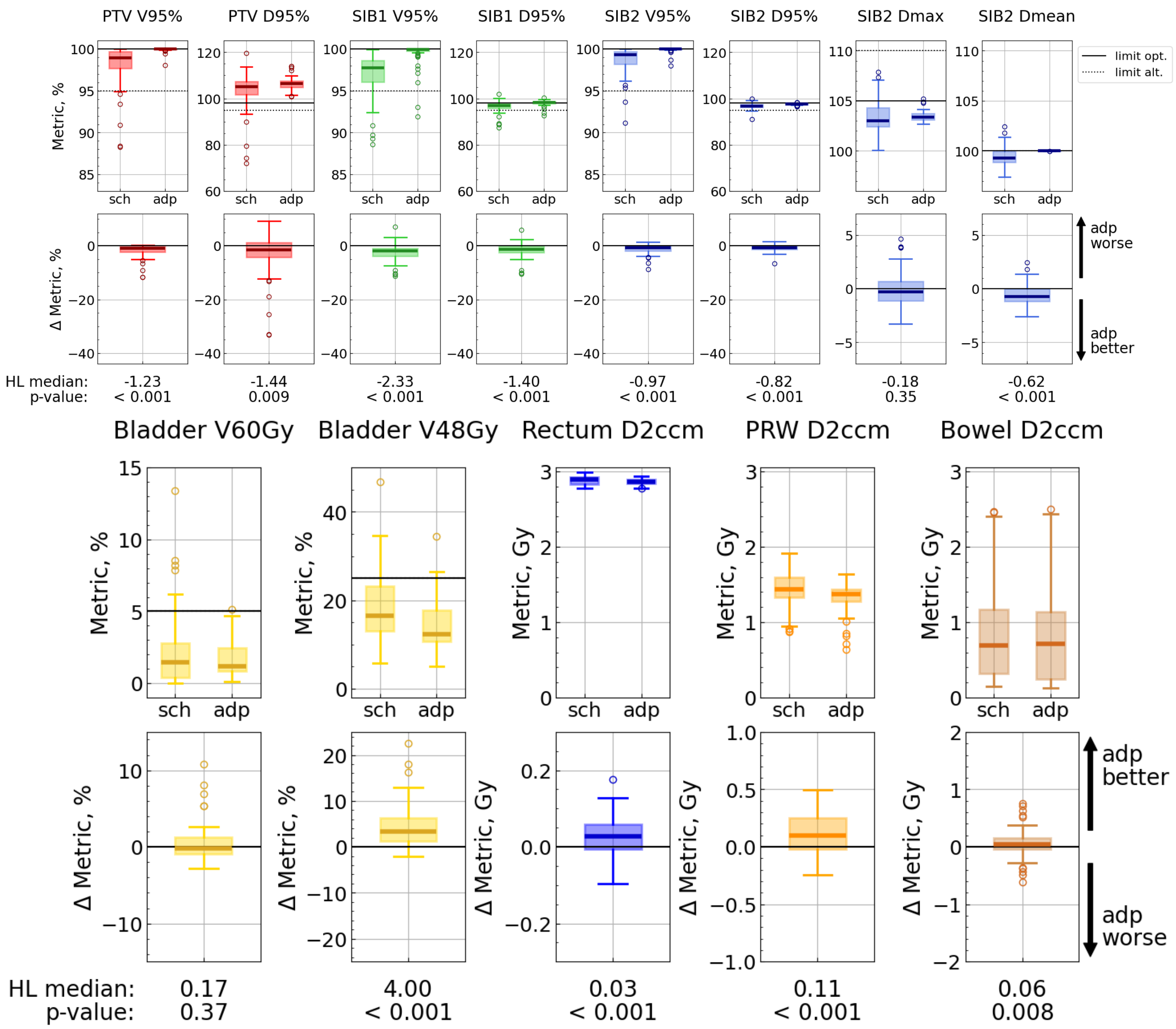

Figure S2. Same as Fig.S1 but for the fractions with prostate contour **smaller**, than the selected threshold $V_{\mathrm{CBCT}} < 0.85 V_{\mathrm{pCT}}$.

2 INCLUDED FRACTIONS

Table S1. Statistical data for target and OAR metrics for scheduled and adapted plans, as well as for difference between metric values obtained with scheduled and adapted plans. It includes Hodges-Lehmann medians, classical first and third quartiles, and p-values obtained with Wilcoxon test. Q1 and Q3 have the same units as the corresponding median. A superior plan ("Sup. plan") is given for each single metric, if the Wilcoxon test resulted in statistical significant difference. Additionally, the percentage of fractions with satisfied alternative goal (column "sat.") is given for each metric (if applicable). Only sessions, in which the prostate contour volume satisfied the selected criterion: $0.85V_{\mathrm{pCT}} \leqslant V_{\mathrm{CBCT}} \leqslant 1.15V_{\mathrm{pCT}}$, are considered in this table.

		Scheduled plan				Adapted plan				Scheduled–adapted			Sup.	
Metric		Median	Q1	Q3	sat.	Median	Q1	Q3	sat.	Median	Q1	Q3	plan	p-val.
PTV	V95%	97.6%	96.4	98.6	84.4	99.995%	99.965	100.000	100.0	−2.32%	−3.61	−1.40	adp	<0.001
	D95%	101.7%	98.3	104.2	84.4	107.6%	106.3	108.7	100.0	−5.97%	−9.58	−3.33	adp	<0.001
SIB1	V95%	93.9%	92.1	95.4	40.8	99.90%	99.72	99.97	99.1	−5.81%	−7.59	−4.23	adp	<0.001
	D95%	93.9%	91.8	95.3	40.8	98.5%	98.1	98.9	99.1	−4.45%	−6.62	−3.05	adp	<0.001
SIB2	V95%	97.1%	95.7	98.2	80.6	99.97%	99.92	99.99	100.0	−2.81%	−4.18	−1.75	adp	<0.001
	D95%	96.0%	95.4	96.5	80.6	97.50%	97.32	97.66	100.0	−1.52%	−2.11	−0.97	adp	<0.001
	Dmean	99.4%	98.9	99.9	27.3	100.003%	99.998	100.007	65.6	−0.60%	−1.07	−0.12	adp	<0.001
	Dmax	104.0%	103.3	104.6	100.0	103.4%	103.2	103.7	100.0	0.48%	−0.22	1.22	adp	<0.001
Bladder	V60Gy	1.40%	0.72	2.55	87.2	1.252%	0.823	2.002	97.6	0.03%	−0.50	0.87		0.67
	V48Gy	13.1%	9.5	17.1	91.2	12.0%	9.3	15.0	97.9	0.70%	−0.48	2.54	adp	<0.001
	V40Gy	18.1%	13.4	23.4	99.3	16.7%	13.1	20.6	99.5	0.96%	−0.55	3.36	adp	<0.001
Rectum	V56Gy	4.9%	3.7	6.3	100.0	5.3%	4.4	6.4	100.0	−0.44%	−1.25	0.44	sch	<0.001
	V52Gy	10.2%	8.4	12.2	100.0	10.8%	9.3	12.4	100.0	−0.62%	−1.67	0.56	sch	<0.001
	V48Gy	14.6%	12.3	17.0	100.0	15.2%	13.3	17.3	100.0	−0.72%	−2.06	0.82	sch	<0.001
	V40Gy	22.6%	19.5	25.9	100.0	23.3%	20.8	25.9	100.0	−0.76%	−2.55	1.34	sch	<0.001
	V32Gy	33.61%	29.64	37.63	97.2	33.79%	30.76	36.94	100.0	−0.40%	−2.87	2.43		0.14
	V24Gy	49.8%	44.6	55.0	96.7	47.9%	44.8	51.5	100.0	1.29%	−2.38	5.36	adp	0.001
	D2ccm	2.853 Gy	2.816	2.892		2.858 Gy	2.836	2.881		−0.007 Gy	−0.042	0.026		0.01
PRW	V37Gy	0.86%	0.10	2.97	86.0	0.075%	0.065	0.300	98.8	0.65%	0.00	2.44	adp	<0.001
	D2ccm	1.39 Gy	1.26	1.52		1.31 Gy	1.22	1.40		0.08 Gy	−0.01	0.18	adp	<0.001
	Dmax	1.93 Gy	1.76	2.13	69.2	1.74 Gy	1.67	1.84	92.4	0.17 Gy	0.05	0.31	adp	<0.001
Bowel	V48Gy	0.012 ccm	0.000	0.135	33.4	0.011 ccm	0.000	0.178	32.9	0.000 ccm	0.000	0.006		0.96
	V40Gy	0.068 ccm	0.000	0.716	43.6	0.049 ccm	0.000	0.713	41.7	0.000 ccm	−0.092	0.033		0.37
	D2ccm	0.69 Gy	0.37	1.14		0.672 Gy	0.364	1.153		0.004 Gy	−0.082	0.093		0.52
	Dmax	1.361 Gy	0.847	1.805	89.1	1.35 Gy	0.82	1.79	90.0	0.004 Gy	−0.116	0.140		0.71
